# Cultivar-Dependent Responses of Eggplant (*Solanum melongena* L.) to Simultaneous *Verticillium dahliae* Infection and Drought

**DOI:** 10.3389/fpls.2018.01181

**Published:** 2018-08-13

**Authors:** Eleni Tani, Dimosthenis Kizis, Emilia Markellou, Ioannis Papadakis, Dimitra Tsamadia, Georgios Leventis, Despoina Makrogianni, Ioannis Karapanos

**Affiliations:** ^1^Laboratory of Plant Breeding and Biometry, Department of Crop Science, Agricultural University of Athens, Athens, Greece; ^2^Laboratory of Mycology, Department of Phytopathology, Benaki Phytopathological Institute, Athens, Greece; ^3^Laboratory of Pomology, Department of Crop Science, Agricultural University of Athens, Athens, Greece; ^4^Laboratory of Vegetable Production, Department of Crop Science, Agricultural University of Athens, Athens, Greece

**Keywords:** eggplant, *Verticillium dahliae*, water stress, combined stress, cultivar, plant growth, plant physiological, molecular responses

## Abstract

Several studies regarding the imposition of stresses simultaneously in plants have shown that plant responses are different under individual and combined stress. Pathogen infection in combination with drought can act both additively and antagonistically, suggesting a tailored-made plant response to these stresses. The aforementioned combination of stresses can be considered as one of the most important factors affecting global crop production. In the present research we studied eggplant responses to simultaneous *Verticillium dahliae* infection and drought with respect to the application of the individual stresses alone and investigated the extent to which these responses were cultivar dependent. Two eggplant cultivars (Skoutari and EMI) with intermediate resistance to *V. dahliae* were subjected to combined stress for a 3-week period. Significant differences in plant growth, several physiological and biochemical parameters (photosynthesis rate, leaf gas exchanges, Malondialdehyde, Proline) and gene expression, were found between plants subjected to combined and individual stresses. Furthermore, plant growth and molecular (lipid peroxidation, hydrogen peroxide, gene expression levels) changes highlight a clear discrimination between the two cultivars in response to simultaneous *V. dahliae* infection and drought. Our results showed that combined stress affects significantly plants responses compared to the application of individual stresses alone and that these responses are cultivar dependent.

## Introduction

Plants in their natural habitats are constantly and most of the time simultaneously exposed to a diverse range of biotic (fungi, bacteria, nematodes, insects, and non cellular pathogens such as viruses and viroids) and abiotic (drought, heat, cold, salinity, heavy metals) stresses. Being sessile, plants have developed specific mechanisms to overcome these challenges and survive. These mechanisms involve overlapping regulatory networks functioning principally at the cellular level and include molecular components that allow plants to perceive environmental changes and pathogen recognition; biochemical changes (such as inorganic ion fluxes, alteration of the redox status and metabolite homeostasis); plant hormones and activated/deactivated protein cascades. These networks participate in stress-signaling mediation as well as in the regulation of local defense responses such as, changes in cell wall and membrane composition and properties, callose deposition, production of reactive oxygen species (ROS), secondary metabolites and osmoprotectants and increase of soluble solutes (Fujita et al., 2006; Atkinson and Urwin, 2012; Kissoudis et al., 2014; Ramegowda and Senthil-Kumar, 2015). These mechanisms also involve systemic signals that prime plant defense to counter subsequent infection attempts and abiotic stresses (Prime-A-Plant Group et al., 2006; Conrath et al., 2015; Nejat and Mantri, 2017) and initiate adaptive responses to abiotic stresses through plant morphological and physiological changes such as changes in root system architecture, cuticular wax composition and leaf water potential (Xiong and Yang, 2003; Enright and Cipollini, 2011; Matus et al., 2014).

Previous evidence from laboratory studies regarding molecular and -omics data analyses (Ma et al., 2006; Seo et al., 2011; Rasmussen et al., 2013; Shaik and Ramakrishna, 2014) as well as field studies (Rizhsky et al., 2004; Mittler, 2006; Mittler and Blumwald, 2010), has shown that there is a synergistic or antagonistic crosstalk between plants responses to combined stress, and that plants respond to a specific combination of stresses in a tailored-made fashion rather than in an additive effect manner. A sequential occurrence of a certain combination of biotic and abiotic stresses may have different impacts, either positive or negative on the plant’s responses regarding pathogen resistance, abiotic stress tolerance, plant performance and fitness costs (Atkinson and Urwin, 2012; Kissoudis et al., 2014; Ramegowda and Senthil-Kumar, 2015; Nejat and Mantri, 2017; Pandey et al., 2017; Zhang and Sonnewald, 2017). These responses do not only depend on the nature of the stresses *per se* (type of stress, combination of stress types), but should be studied in relation to stress intensity, the order of occurrence (i.e., pathogen infection prior to an abiotic challenge or acclimation response followed by biotic stress), and the species genetic background (Kissoudis et al., 2014; Nejat and Mantri, 2017). The challenge to study the combinatory effect of multiple environmental stresses on plants performance becomes more evident, taking into consideration the effects of a climate change scenario on future agriculture needs. Under such environmental conditions plant–pathogen–abiotic stress interactions are drastically affected.

Drought stress is undoubtedly one of the most important of several, environmental factors that can greatly influence the productivity of crop plants around the world (Reddy et al., 2004). Plants respond to drought through multiple mechanisms such as altering shoot/root ratio (Bootraa et al., 2010), reducing plant growth rates (Reddy et al., 2004) and increasing water use efficiency (WUE) (Sivamani et al., 2000; Bootraa et al., 2010). On the other hand, plant pathogenic fungi are responsible for serious yield losses on an annual basis (Savary et al., 2012). *Verticillium dahliae* Kleb., is a widely distributed soilborne fungus, causing destructive vascular wilt to a wide range of herbaceous and woody plant hosts and severe yield losses (Pegg and Brady, 2002; Daayf, 2015). Since there are no effective chemical treatments to control the disease, management strategies encounter preventive measures (Blok et al., 2000), biological control regimes (Mercado-Blanco, 2012; Angelopoulou et al., 2014; Markakis et al., 2016) and use of cultivars that tolerate the disease to a certain extent (López-Escudero et al., 2004; Zhang et al., 2015). Many biochemical responses induced by drought are similar to those induced by pathogen attack, including increases in proline content (Farooq et al., 2009; Verslues and Sharma, 2010; Liang et al., 2013), ROS formation (Borden and Higgins, 2002; Degara et al., 2003), and upregulation of gene expression (Ramirez et al., 2009; Asano et al., 2012).

Previous publications show that in the cases of combined stresses, there is either a positive or negative effect of the preceding stress (i.e., drought or a specific pathogen attack) on plant responses toward the succeeding stress. In general drought influences negatively the plants tolerance to pathogen infection, causing detrimental effects to plants pathogen resistance (Mayek-Perez et al., 2002; Wang et al., 2009). On the other hand, increased resistance of barley plants to barley powdery mildew under drought conditions was observed through shared defense responses (Wiese et al., 2004). Additionally, tomato plants growth under drought conditions showed enhanced resistance to *Botrytis cinerea* and *Oidium neolycopersici* (Achuo et al., 2006). Pathogen infection often results in lower photosynthetic rates and reduction of WUE (Grimmer et al., 2012) and increases the susceptibility to drought stress (Audebert et al., 2000). Nevertheless, *Arabidopsis* plants infected with *V. dahliae* showed increased drought tolerance due to new xylem formation (Reusche et al., 2012). Furthermore, beneficial microbes such as arbuscular mycorrhizae and certain types of bacteria were shown to affect positively drought tolerance (Aroca et al., 2008; Kohler et al., 2008).

Eggplant (*Solanum melongena* L.) is one of the most important vegetable crops, especially for the Mediterranean basin, after potato (*Solanum tuberosum*) and tomato (*Lycopersicon esculentum*) (Rotino et al., 2014). Eggplants are very susceptible to many abiotic and biotic limiting factors throughout their entire biological cycle. They are very sensitive to water deficit, as they need substantial amount of water for their growth and development (Fu et al., 2013) and are susceptible to important fungal pathogens, such as the *Fusarium oxysporum* and *V. dahliae* (Collonnier et al., 2001), which cause significant crop damage. Therefore, one of the main objectives of eggplant breeding programs is the improvement of resistance to biotic and abiotic stresses. As eggplant is one of the most important vegetables for the Mediterranean region, and climate change will enforce the frequency as well as the intensity of combined stresses, it is of great importance to address the issues of climate-resilient vegetables.

In the present work we studied eggplant responses to the combined application of two main stress factors that affect its cultivation (*V. dahliae* and drought), compared to the application of the individual stresses alone. In addition, we investigated the extent to which these responses to combined stress are cultivar dependent. We showed that combined stress affects significantly the plants physiological and biochemical responses compared to application of individual stresses alone and that cultivar dependent plant growth significant differences are also affected by the type and combination of stresses applied. Furthermore, we showed that the expression of specific eggplant abiotic and biotic stress marker genes is induced by both drought and *V. dahliae* infection and that combined stress strongly enhances the expression of biotic stress marker genes in the leaves and roots of both eggplant cultivars.

## Materials and Methods

### Plant Material and Treatments

Two of the most common Greek eggplant cultivars, “Skoutari” and “EMI” with intermediate resistance to *V. dahliae* (Bletsos, unpublished results) were selected for this study. The cultivars were provided by the Agricultural Research Center of Northern Greece, ELGO-Demetra. Seeds were surface sterilized in 1.25% sodium hypochlorite for 20 min, rinsed three times in sterile distilled water, sown in sterile Klasmann-TS2 soil (Klasmann-Deilmann, Geeste, Germany) distributed in 50-ml QP Standard plastic pot trays (Agrohoum, Athens, Greece) and allowed to germinate in darkness. Plantlets were grown in a glasshouse under controlled conditions of 24°C, 70–80% RH, 16 h light – 8 h dark photoperiod, maximum light intensity 1110 μmol photons m^-2^ s^-1^. The eggplant seedlings were transplanted to 10 cm diameter pots when they were 7 weeks old at the stage of four true leaves. The selected seedlings were artificially inoculated either with a 10^6^ mL^-1^ *V. dahliae* conidial suspension or with water for mock controls, just before transplantation, according to the dip-root method reported by Cappelli et al. (1995). The virulent BPIC 2681 *V. dahliae* isolate from eggplant, maintained in the BPI official collection of fungi, was used for inoculation. The seedlings were then watered and fertilized with Nutri-Leaf 20:20:20 (Miller Chemical & Fertilizer, LLC, Hanover, PA, United States) every week. Pots were arranged in a complete randomized design. Drought stress regimes were initiated the day the first wilting symptoms appeared, which was 17 days post inoculation (DPI) with the pathogen. For a time-interval of 3 weeks, four groups of plants were formed, each subjected to a specific treatment: (a) plants watered up to 100% of soil field capacity (FC) that served as a control, (b) plants infected with *V. dahliae* and watered up to 100% of FC, (c) plants watered up to 25% of FC, and (d) plants infected with *V. dahliae* and watered up to 25% of FC.

### Leaf and Root Material Sampling

For all measurements and analyses performed, each plant constituted a single biological replicate. Leaf and root material sampling was performed during the last day of the treatments applied. For the determination of the H_2_O_2_, Malondialdehyde (MDA), and Proline concentrations and for RNA extraction, leaf and root material were collected from individual plants for each of the different treatments. For the leaf material the first, third, and forth fully developed leaves from the shoot apex were collected. The whole root system was collected after washing out the soil from the rhizosphere for a time interval of up to 3 min and using tap water previously acclimated at glasshouse controlled temperature conditions. Samples were briefly dried, immediately frozen in liquid nitrogen and stored at -80°C before subsequent use.

### Plant Growth Parameters

The length of the stems from the base to the tip and the number of leaves were measured in each plant at an interval of 3 days through the duration of the experiment. Relative Growth Rate (RGR) respect to stem elongation was estimated as follows: RGR = [(T2-T1)/t]⋅1/T2, where T1 and T2 represent the stem length at the beginning and at the end of a time (t), respectively. Root length was measured at the end of the experiment. Fresh leaves, stems and roots were harvested, and oven dried at 85°C for 48 h to determine the dry weights.

### Leaf Gas Exchange Parameters

Net photosynthetic rate (*P*_N_), stomatal conductance (g_s_), transpiration rate (*E*), and intercellular CO_2_ concentration (*C*i), were measured twice per plant per treatment, using both the third and forth fully developed leaves from the shoot apex. The measurements were carried out with the Li-6400XT (Li-COR, Lincoln, NE, United States) portable photosynthesis measuring system under steady light intensity (800 μmol m^-2^ s^-1^) and CO_2_ concentration (400 mg l^-1^), while leaf temperature ranged between 27.5 and 29.8°C. WUE was computed as the ratio between *P*_N_ and *E* of each one measurement. Similarly, the carbon dioxide use efficiency and the ratio between net photosynthetic rate and stomatal conductance were calculated by dividing the *P*_N_ values with *C*i or g_s_ values of the recorded at each one measurement, respectively.

### Lipid Peroxidation and Hydrogen Peroxide

Leaf tissue (250 mg) was homogenized in 10 ml 0.1% trichloracetic acid (TCA) at 4°C. After centrifugation at 4000 rpm for 15 min, the supernatant was used for the determination of both lipid peroxidation level and H_2_O_2_ concentration. Lipid peroxidation was measured as MDA content determined by reaction with 0.5% 2-thiobarbituric acid (TBA) in 20% TCA (w/v). The concentration of MDA was calculated from the difference of the absorbance at 532 and 600 nm using the extinction coefficient of 155 mmol^-1^ cm^-1^ (Heath and Packer, 1968). Hydrogen peroxide was also measured spectrophotometrically, after reaction with potassium iodide (KI). The reaction mixture consisted of 0.5 mL 0.1% trichloroacetic acid (TCA), leaf extract supernatant, 0.5 mL of 0.1 M potassium-phosphate buffer (pH 7) and 1 mL 1 M KI (w/v) reagent. The reaction color was developed for 45 min in darkness and absorbance was measured at 390 nm. The amount of hydrogen peroxide was calculated using a standard curve prepared with eight known concentrations of H_2_O_2_.

### Proline Concentration

Proline content in root and leaf tissues was measured via reaction with ninhydrin (Bates et al., 1973). For colorimetric determinations, a solution of proline, ninhydrin acid and glacial acetic acid (1:1:1) was incubated at 90°C for 1 h. The reaction was then cooled in an ice bath. The chromophore was extracted using 2 ml of toluene and its absorbance at 520 nm was determined by a BioMate spectrophotometer (Thermo Fisher Scientific, Waltham, MA, United States).

### Primer Sequences

*Solanum melongena* EST sequences were retrieved after homology search from GenBank and Sol Genomics Network. Primers (**Table 1**) for quantitative real time PCR experiments were designed using the OligoPerfect^TM^ Designer (Thermo Fisher Scientific, Waltham, MA, United States), following standard primer design criteria. Primers were tested in end-point and real time PCR reactions using 10-fold dilutions of *S. melongena* genomic DNA ranging from 50 ng to 50 fg.

**Table 1:** Primers sequences used for the gene expression study.

Primer name	Sequence 5′ to 3′	Gene target	GenBank/Sol Genomics Network ID	Amplicon length (bp)	Reference
Sm-AREB-F	GGGATGGTTGGTATCGCTGA	*AREB*	Sme2.5_12232.1_g00002.1	100	This study
Sm-AREB-R	CTCCAGCCCCTAAACCTACC				
Sm-CBF1-F	TGGGTTTGCGAAGTCAGAGA	*CBF*1	Sme2.5_16389.1_g00001.1	121	This study
Sm-CBF1-R	CAGAACGGCCCCTTAATGCT				
Sm-NAC-F	GAGCACCTTCCTCCTGGATT	*NAC*	Sme2.5_15135.1_g00001.1	147	This study
Sm-NAC-R	AGGCAATTCCCAAGGGTCG				
Sm-PR1-F	GTGGGTCGATGAGAAGCAAT	*PR*1	AB222697.1	92	This study
Sm-PR1-R	TACGCCACACCACCTGAGTA				
Sm-PR5-F	CAAACACCCTGGCTGAATACG	*PR*5	Sme2.5_30700.1_g00001.1	113	This study
Sm-PR5-R	ACTAGGATTGGTCGGTGCAA				
Sm-LOX-F	GGAGGGATCAAACTTCCTCA	*LOX*	AB244527.1	101	This study
Sm-LOX-R	ATTCCTTCACCGTCTGTTCG				
Sm-Actin-F	ACCACAGCTGAGCGAGAAAT	*ACTIN*	JX524155.1	133	Zhou et al., 2014
Sm-Actin-R	GACCATCGGGAAGCTCATAG				

### cDNA Synthesis and Relative Gene Expression Study

Total RNA was extracted using the TRI Reagent (Sigma-Aldrich, St. Louis, MI, United States) reagent following the instructions of the manufacturers. cDNA was synthesized using the PrimeScript^TM^ RT reagent Kit with gDNA Eraser (Takara Bio, Shiga, Japan) and 1 μg of total RNA according to the manufacturer’s instructions. The Applied Biosystems 7500 Fast Real-Time PCR system (Applied Biosystems, Foster City, CA, United States) was used for real time PCR assays. Samples were prepared using the KAPA SYBR FAST qPCR kit 2X master mix (KAPA Biosystems, Inc., Boston, MA, United States), 200 nM of each primer and either 1 μl of cDNA template (1/20th of cDNA reaction volume) or RNase free-H_2_O for non-template controls, in a final reaction volume of 15 μl. Four individual biological replicates for each of the different treatments were assayed per RT-PCR run, in two individual runs considered as technical replicates. Non-reverse transcribed samples were also assayed in real time PCR runs prior to sample analyses. For all target genes assayed the PCR amplification thermal profile used consisted of one initial denaturation cycle of 3 min at 95°C followed by 40 cycles of 3 s at 95°C and 20 s at 60°C. Melting curves were programmed as 15 s at 95°C, 15 s at 60°C, 20 min slow ramp, and 15 s at 95°C. The data for the dissociation curve were captured during this slow ramp, and the melt curve was visualized using the ABI PRISM 7900 software. The relative gene expression was determined with the comparative Ct method (Livak and Schmittgen, 2001), calculating the mean threshold cycle (Ct) values of the target and endogenous control genes for four individual biological replicates of both technical replicate runs.

### Statistical Analysis

Analysis of Variance (three-way ANOVA) was carried out with *V. dahliae* infection, water stress level and cultivars as fixed factors (sample size: 24 or 32 plants). Differences between means were assessed using Fisher’s Protected least significant difference (LSD) (*P* < 0.05). The statistical package used for analyzing the data was GENSTAT 10th edition. Normality was checked by examining residual plots produced which provide a way of checking the normality and the equal variance assumptions of the ANOVA and the Shapiro–Wilk test. The data was found to be normally distributed. In addition, Analysis of Variance (one-way ANOVA) was carried out for the parameters presented in **Figures 2**, **3** to test differences between treatments within each cultivar. Differences between means were assessed using Student Newman Keuls test (*P* < 0.05).

## Results

### *Verticillium dahliae*, Drought Stress and Eggplant Cultivars Double and Triple Interactions Significantly Affect the Plant Relative Growth Rate and the Shoot to Root Fresh Weight Ratio

**FIGURE 1:**
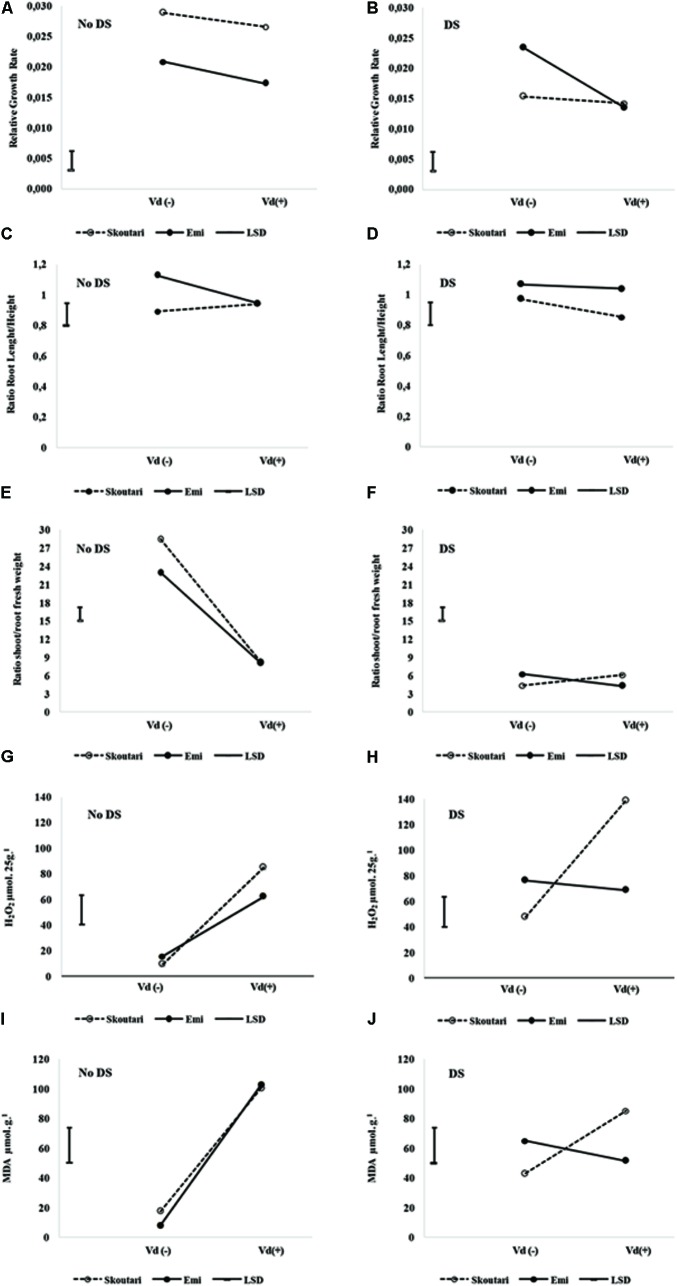
Three-way interaction plots of RGR **(A,B)**, root to stem length ratio **(C,D)**, shoot to root fresh weight ratio **(E,F)**, H_2_O_2_ **(G,H)**, MDA **(I,J)** and Proline **(K,L)** concentrations, on two eggplant cultivars (Skoutari and Emi), at *V. dahliae* infection and drought stress. Vd (±): *Verticillium dahliae*, DS: drought stress.

The significance of the pathogen, water stress and cultivars main effects and their double and triple interactions on RGR, root to stem length ratio and shoot to root fresh and dry weight ratios, are presented in **Tables 2** and **3**. Triple interactions are further presented in **Figure 1** by creating separate plots which illustrate the interaction of cultivar and *V. dahliae* stress (Vd-/+) at normally irrigated (No DS) and water stressed (DS) plants. In the graphs, the mean value of each parameter assesses/measured and the LSD values are presented.

**Table 2:** Effect of, and interactions between, *Verticillium dahliae*, drought stress and cultivars on developmental, physiological and biochemical parameters eggplants (3-factor ANOVA).

Factors		RGR (cm⋅day^-1^ cm^-1^)	Root/Stem length ratio	Shoot/Root fresh weight ratio	Shoot/Root dry weight ratio	WUE (μmol CO_2_⋅mmol H_2_O^-1^)	*P*_N_/g_s_ (μmol CO_2_⋅mol H_2_O^-1^)	H_2_O_2_ (μmol⋅25 g^-1^)	MDA (μmol g^-1^)	Proline (mg⋅100 g^-1^)
***Verticillium dahliae***	No	0.020	1.01	**15.51**	7.83	5.67	132.11	**42.9**	57.1	194.5
(*n* = 24 or 32)	Yes	0.020	0.95	**6.65**	7.32	6.03	146.24	**83.0**	61.1	222.1
**Drought stress**	No	**0.023**	0.98	**16.90**	**8.29**	**4.28**	**97.96**	**37.3**	**33.2**	**168.3**
(*n* = 24 or 32)	Yes	**0.017**	0.98	**5.26**	**6.86**	**7.41**	**180.39**	**88.6**	**84.9**	**248.2**
**Cultivars**	Skoutari	**0.024**	**0.91**	**11.78**	7.86	5.72	139.12	**70.3**	61.4	**188.3**
(*n* = 24 or 32)	EMI	**0.016**	**1.04**	**10.38**	7.30	5.99	139.23	**55.6**	56.7	**228.3**

**ANOVA results (*p*-value)**										
*Verticillium dahliae* (Vd)		NS	NS	**<0.001**	NS	NS	NS	**<0.001**	NS	NS
Drought stress (DS)		**<0.001**	NS	**<0.001**	**<0.001**	**<0.001**	**<0.001**	**<0.001**	**<0.001**	**<0.001**
Cultivars (C)		**<0.001**	**<0.001**	**0.017**	NS	NS	NS	**0.015**	**NS**	**0.022**
Vd x DS		**<0.001**	NS	**<0.001**	NS	**<0.001**	**<0.001**	NS	**<0.001**	**<0.001**
Vd x C		**0.002**	NS	NS	NS	NS	NS	NS	NS	NS
DS x C		**0.047**	NS	**0.016**	NS	NS	NS	**<0.001**	NS	**0.005**
Vd x DS x C		**0.019**	**0.033**	**<0.001**	NS	NS	NS	**0.005**	**0.006**	**<0.001**

*The values represent means; NS, not significant; RGR, Relative Growth Rate; WUE, Water Use Efficiency; *P*_*N*_, Photosynthesis rate; g_*s*_ Stomatal conductivity; MDA, Malondialdehyde. Bold values indicate statistically significant differences*.

**Table 3:** Effect of *Verticillium dahliae* infection and drought stress on eggplant developmental, physiological and biochemical parameters.

	RGR (cm⋅day^-1^⋅cm^-1^)	Shoot/Root fresh weight ratio	WUE (μmol CO_2_⋅mmol H_2_O^-1^)	*P*_N_/g_*s*_ (μmol CO_2_⋅mol H_2_O^-1^)	MDA (μmol g^-1^)	Proline (mg⋅100 g^-1^)
no DS – no Vd	0.025 d	25.70 c	3.46 a	74.25 a	12.6 a	26.1 a
DS – no Vd	0.015 a	5.31 a	7.87 c	189.98 c	101.6 c	362.9 d
no DS – Vd	0.022 c	8.10 b	5.11 b	121.66 b	53.57 b	310.6 c
DS – Vd	0.019 b	5.20 a	6.96 c	170.81 c	68.2 b	133.6 b
*p*-value	**<0.001**	**<0.001**	**<0.001**	**<0.001**	**<0.001**	**<0.001**
LSD	0.0022	1.61	1.02	23.34	16.73	47.51

*The values represent means; RGR, Relative Growth Rate; WUE, Water Use Efficiency; PN, Photosynthesis rate; gs Stomatal conductivity; MDA, Malondialdehyde; DS, Drought Stress; Vd, *Verticillium dahlia*. Bold values indicate statistically significant differences*.

Drought stress and cultivars as single factors had significant effect on RGR (**Table 2**). *V. dahliae*, water stress and cultivars imposed together had also significant effect on RGR as indicated in all double and triple interactions, presented in **Table 2** and **Figures 1A,B**. As presented in **Table 3**, for the *V. dahliae* and drought stress combination (irrespective of the cultivar) RGR values for all plant treatments were significantly reduced compared to the control (0.025 cm day^-1^ cm^-1^). Plants under combined stress had an intermediate RGR value (0.019 cm day^-1^ cm^-1^) with respect to *V. dahliae* infected (0.022 cm day^-1^ cm^-1^) and drought-stressed (0.015 cm day^-1^ cm^-1^) alone.

Cultivars was the only significant main factor to affect the plants root to stem length ratio (mean values of 0.91 and 1.04 for Skoutari and EMI cultivars, respectively). Triple interaction of cultivar with the two stress types were also found to be statistically significant (**Table 2**). All three factors affected significantly the shoot to root fresh weight ratio, as main factors and in their double and triple interactions except the interaction between *V. dahliae* and cultivars (mean values and significance levels are presented in **Table 2** and **Figures 1C,D**). As presented in **Table 3** for the *V. dahliae* and water stress interaction, the ratio was much lower in infected plants (8.10) respect to the control (25.70) and was reduced further in drought stressed plants (5.31) and plants under combined stress (5.20). The cultivars significant effect with the *V. dahliae* and drought stress interaction on the shoot to root fresh weight ratio is presented in **Figures 1E,F**. The shoot to root dry weight ratio was affected significantly only by drought stress (mean values of 8.29 and 6.86 for control and stressed plants, respectively, **Table 2**). The values of the dry weight ratio for the *V. dahliae* infected and drought stressed plants were higher respect to their corresponding fresh weight ratio values.

### Similar Reduction in Photosynthesis Rate and Leaf Gas Exchange Parameters in Plants Under Drought and Combined Stress

**FIGURE 2:**
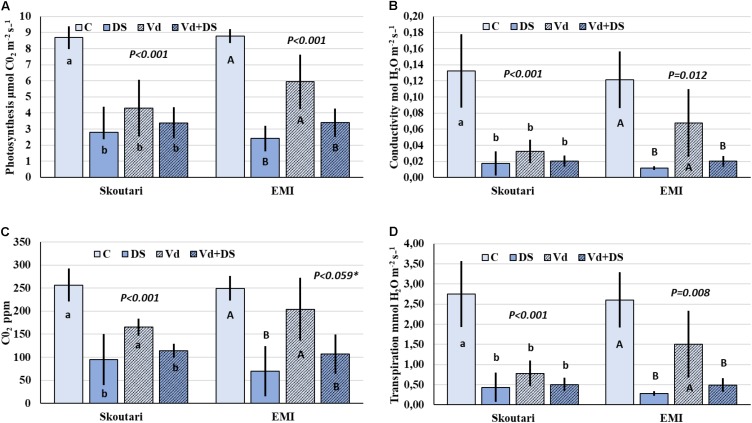
Photosynthesis rate and leaf gas exchange parameters in eggplant cultivars subjected to abiotic, biotic and combined stresses. The net photosynthetic rate (*P*_N_), stomatal conductance (g_*s*_), intercellular CO_2_ concentration (*C*i) and transpiration rate (*E*) (**A–D**, respectively), were measured in eggplant cultivars under individual and combined stress. C, control; DS, drought stress; Vd, *Verticillium dahliae*; Vd+DS, Combined stress. Bars represent standard deviation. Means within the same cultivar with different lower (Skoutari) or upper (EMI)-case letters are significantly different according to Student Newman Keuls test (*P* < 0.05 or *P* < 0.1^∗^).

Photosynthesis rate (*P*_N_), and leaf gas exchange parameters (g_s_, *E*, and *C*i), were reduced in both cultivars in all stresses applied respect to the untreated control (**Figures 2A–D**). Furthermore, the same pattern regarding the reduction levels was observed in between stress treatments. In both cultivars, all parameters measured were affected more (with respect to the mean values) by drought stress and less by *V. dahliae* infection, whereas the combined stress resulted to intermediate mean values respect to those of drought stressed and pathogen infected plants alone (**Figures 2A–D**). For the Skoutari cultivar, statistically significant differences for the three out of four parameters tested were observed between treated plants (irrespective the type of stress) and untreated controls. For the EMI cultivar, statistically significant differences were observed for all parameters tested between plants treated either with drought or combined stress in comparison to control or *V. dahliae* infected plants (**Figures 2A–D**).

A statistical analysis of the pathogen, drought stress and cultivar main effects as individual factors and the significance of their double and triple interactions, on WUE and Photosynthesis rate/Conductivity ratio (*P*_N_/g_s_) are presented in **Tables 2**,**3**. Drought stress had a significant effect on both ratios, while *V. dahliae* and cultivars had no significant effect on either ratio (**Table 2**). With respect to *V. dahliae*, drought stress and cultivar double interactions, combined stress (irrespective the cultivar) was the only one to affect significantly both ratios (**Tables 2**,**3**). Specifically, WUE increased in *V. dahliae* infected plants (5.11 μmol CO_2_⋅mmol H_2_O^-1^) compared to the control (3.46 μmol CO_2_⋅mmol H_2_O^-1^) and doubled in plants under drought and combined stresses (7.87 and 6.96 μmol CO_2_⋅mmol H_2_O^-1^, respectively) (**Table 3**). The *P*_N_/g_s_ ratio followed the same pattern as WUE and increased in *V. dahliae* infected plants (121.66 μmol CO_2_ mol H_2_O^-1^) with respect to the control (74.25 μmol CO_2_⋅mol H_2_O^-1^) and was more than double in plants under drought and combined stresses (189.98 and 170.81 μmol CO_2_⋅mol H_2_O^-1^, respectively) (**Table 3**). The combination of cultivar, *V. dahliae* and drought stress had no significant effect on WUE and *P*_N_/g_s_ ratio as presented in **Table 2**.

### Determination of H_2_O_2_, MDA, and Proline Concentrations in Leaves of Plants Treated With Individual and Combined Stresses

**FIGURE 3:**
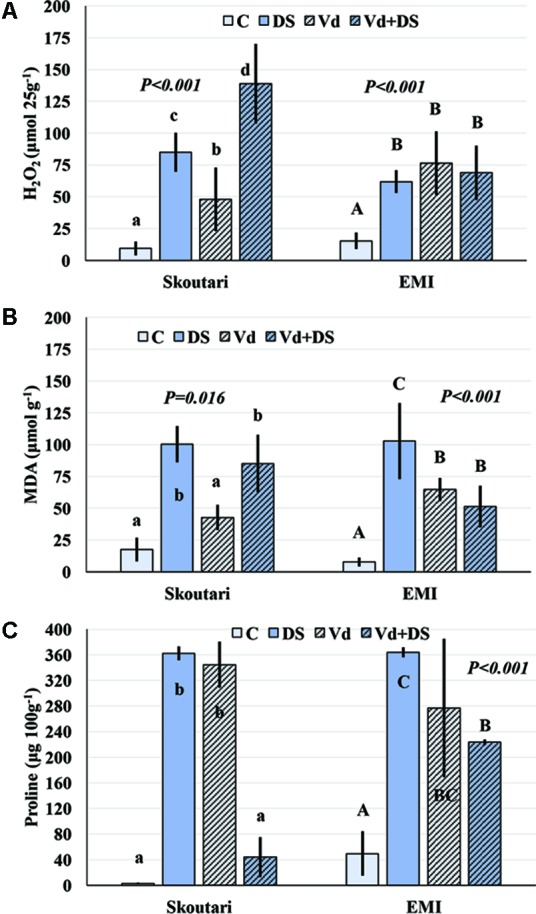
H_2_O_2_, MDA, and Proline concentration alterations in eggplant cultivars subjected to abiotic, biotic and combined stresses. The H_2_O_2_, MDA, and Proline concentration (**A–C**, respectively) were measured in eggplant cultivars under individual and combined stress. C, control, DS, drought stress, Vd, *Verticillium dahliae*, Vd+DS, Combined stress. Bars represent standard deviation. Means within the same cultivar with different lower (Skoutari) or upper (EMI)-case letters are significantly different according to Student Newman Keuls test (*P* < 0.05).

The concentration of H_2_O_2_ in leaves increased after application of either individual or combined stresses with respect to the untreated control plants, however, with a different mode for the two cultivars (**Figure 3A**). The highest H_2_O_2_ concentration (138.81 μmol⋅25 g^-1^ of tissue fresh weight) was observed in Skoutari plants under combined stress. In the same cultivar, H_2_O_2_ mean levels in individually drought stressed or *V. dahliae* infected plants were at 85.00 and 47.91 μmol⋅25 g^-1^, respectively, higher than the untreated control (9.36 μmol⋅25 g^-1^). For Skoutari plants statistical differences were observed between all treatments and the control. On the other hand, H_2_O_2_ in leaves of EMI treated plants was similar with mean values of 68.81, 61.90, and 76.45 μmol⋅25 g^-1^, for plants under combined stress, drought stress or *V. dahliae* infection, respectively. They all are statistically different from the untreated control (15.36 μmol⋅25 g^-1^).

Malondialdehyde concentrations varied between treatments in both cultivars (different statistical significance in each cultivar), as seen in **Figure 3B**. The highest mean values were observed in drought stressed plants of both cultivars (100.38 and 102.83 μmol g^-1^ for Skoutari and EMI, respectively), whereas, *V. dahliae* infected plants showed lower MDA mean values (42.71 and 64.77 μmol g^-1^ for Skoutari and EMI, respectively) compared to drought stressed plants. In plants subjected to combined stress, MDA ranged at the same levels as drought stressed plants (mean value of 85.03 μmol g^-1^) for Skoutari, however, was lower (mean value of 51.35 μmol g^-1^) for EMI. In untreated controls MDA mean values were 17.54 and 7.74 μmol g^-1^ for Skoutari and EMI, respectively.

As presented in **Figure 3C**, proline concentration was increased in all treated samples. A high increase was observed respect to the controls (mean values of 2.7 mg⋅100 g^-1^ and 49.5 mg⋅100 g^-1^ for Skoutari and EMI, respectively) in drought stressed (362.1 mg⋅100 g^-1^ and 363.7 mg⋅100 g^-1^ for Skoutari and EMI, respectively) and *V. dahliae* infected plants (344.4 mg⋅100 g^-1^ and 276.7 mg⋅100 g^-1^ for Skoutari and EMI, respectively). It was, however, reduced in plants subjected to combined stress (43.9 mg⋅100 g^-1^ for Skoutari and 223.3 mg⋅100 g^-1^ for EMI). Although, a similar pattern of the plant’s response to the stresses applied is observed for both cultivars, statistically significant differences vary in between stress types for each cultivar.

The statistical analysis of the pathogen, drought stress and cultivar main effects as individual factors and the significance of their double and triple interactions, on H_2_O_2_, MDA, and Proline concentrations are presented in **Tables 2**, **3**. *V. dahliae* had a significant effect on H_2_O_2_ only, while drought stress influenced all three parameters tested. Cultivar as single factor had a significant effect on both H_2_O_2_ and Proline (**Table 2**). The combined *V. dahliae* and drought stress (irrespective the cultivar) had significant effect on MDA and Proline concentrations as indicated in **Tables 2**, **3**. Specifically, MDA concentrations were significantly increased in all treated samples respect to the control, with the highest mean value observed (**Table 3**) in drought stressed plants (101.6 μmol g^-1^). MDA concentration was reduced to nearly half in *V. dahliae* infected plants (53.57 μmol g^-1^) and had an intermediate value (68.2 μmol g^-1^) for plants under combined stress, though not statistically different (LSD, 16.73) from the former. Proline concentrations (**Table 3**) were significantly increased in plants subjected to individual stress (mean values of 362.9 and 310.0 mg⋅100 g^-1^ for drought stressed and *V. dahliae* infected plants, respectively), respect to the control (26.1 mg⋅100 g^-1^). Plants under combined stress had an intermediate mean value (133.6 mg⋅100 g^-1^) though statistically different from the control (LSD, 47.51). No significant interaction of *V. dahliae* and variety was observed for all three parameters tested, while the drought stress and variety interaction were only significant for H_2_O_2_ and Proline (**Table 2**). The cultivar, *V. dahliae* and drought stress triple interaction had a combined effect on H_2_O_2_, MDA, and Proline (**Table 2**) with significant differences between plants of the two cultivars observed under combined stress (**Figures 1G–L**).

### Combined *V. dahliae* and Drought Stress Enhances the Expression of Biotic Stress Marker Genes

**FIGURE 4:**
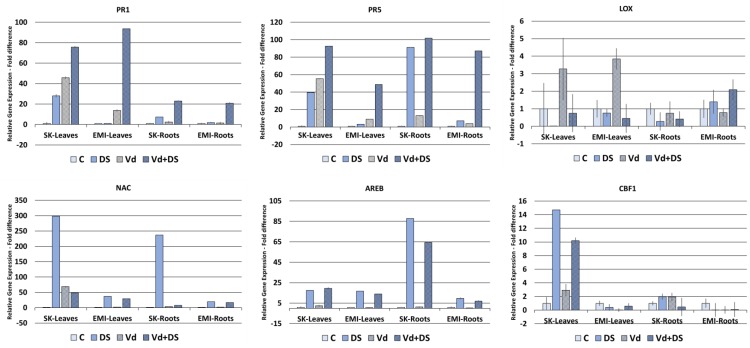
Relative gene expression of the *PR*1, *PR*5, *LOX*, *NAC*, *AREB*, and *CBF*1 marker genes in leaves and roots of eggplant plants subjected to abiotic, biotic, and combined stresses. The relative expression of selected marker genes was measured in eggplant cultivars under individual and combined stress. C, control; DS, drought stress; Vd, *Verticillium dahliae*; Vd+DS, Combined stress. Bars represent standard error.

**Figure 4** shows the gene expression fold differences of selected marker genes for the two cultivars subjected under individual and combined stresses. For the *PR*1 and *PR*5 biotic stress marker genes a basal level of expression was detected, as for the abiotic stress marker genes, in untreated controls in all cases. Expression of the two genes was induced in *V. dahliae* infected plants as expected and in plants under drought stress, in leaves and roots of both cultivars. A strong upregulation of gene expression was observed in plants under combined stress compared to plants either under biotic or drought stress alone, with the highest values (fold changes respect to untreated controls) observed in leaves with respect to roots of both cultivars for the *PR*1 gene (76-fold and 94-fold for the Skoutari and EMI cultivars, respectively) and in roots with respect to leaves for the *PR*5 gene (102-fold and 87-fold for the Skoutari and EMI cultivars, respectively). The expression of the *LOX* gene did not vary significantly between the different treatments and the control for both cultivars and organ types.

The *NAC* marker gene was significantly induced in drought treated plants showing the highest values (fold changes respect to untreated controls) in both tissues of the Skoutari cultivar (298-fold and 237-fold, respectively). Significant induction was also observed in plants subjected to combined stress, however, fold changes ranged at the same levels as of drought induced plants for the EMI cultivar in both leaves and roots (29-fold and 37-fold for leaves and 17-fold and 19-fold for roots, respectively), but where significantly downregulated compared to drought induced plants for the Skoutari cultivar (48-fold and 298-fold for leaves and 8-fold and 237-fold for roots, respectively). For the *AREB* marker gene significant induction of expression was observed in plants treated with drought and plants under combined stress. In both cases the levels of fold differences did not vary between the two types of treatment irrespectively the cultivar or type of organ with the highest values observed in Skoutari roots (88-fold and 65-fold for drought and combined stress, respectively). Significant gene expression induced by biotic stress alone was observed only for *NAC* in Skoutari leaves (69-fold respect to untreated control). Significant differences in the expression of *CBF*1 were observed only in Skoutari leaves. Expression was induced by all types of treatments with the highest fold differences observed in drought and combined stresses (15-fold and 10-fold compared to untreated control, respectively).

## Discussion

A limited number of studies regarding the imposition of stresses simultaneously in plants revealed that plants respond in a different manner under individual and combined stress (Xu et al., 2008; Atkinson et al., 2013; Prasch and Sonnewald, 2015; Gupta et al., 2016). The combination of two stresses (abiotic–abiotic or abiotic–biotic) does not always have a negative effect on plants. Reports have shown that drought in combination with pathogen infection can act both additively and antagonistically. This combination can be considered as one of the most important stress combinations affecting global crop production, given the number of reports of plant diseases caused by drought stress and the frequency of water stress incidences. Pathogens that induce wilt, such as *V. dahliae*, impose a physiological drought stress effect in plants by blocking the xylem (Genin, 2010). When wilt disease coincides with drought, a new net effect of combined stress could be expected that is different from the individual stresses (Abd El-Rahim et al., 1998; Choi et al., 2013; Sinha et al., 2016). To study this assumption, the impact of the combined occurrence of *V. dahliae* and drought was compared with the impact of the two stressors imposed separately, by measuring several morphological, physiological and biochemical parameters. The simultaneous imposition of drought and a vascular pathogen is often found to reduce several morphological and physiological parameters such as the plant height, total leaf area and transpiration (Pennypacker et al., 1991; Abd El-Rahim et al., 1998; Choi et al., 2013; Sinha et al., 2017).

Our results indicate that the concurrent effect of pathogen and drought stresses is different in each cultivar compared to the net effect of drought stress and *V. dahliae* applied individually. Similarly, the effect of drought and *Verticillium* wilt on eggplant cultivars had been studied under field conditions (Bletsos et al., 1999) and showed that different irrigation regimes in combination with *Verticillium* wilt significantly affected fruit quantitative characteristics of different eggplant cultivars.

Specifically, when drought and pathogen stress occurred simultaneously, they caused a significant decrease in RGR. Plant growth reduction can be the outcome of a pathogen infection (Johnstone et al., 2005). Results by Buhtz et al. (2017) demonstrated that *V. dahliae* infection greatly compromised plant growth of tomato plants. Nevertheless, this reduction can limit the water requirements of the plant thus improving plant survival during the simultaneous imposition of drought as shown in other studies (Xu et al., 2008). Interestingly our results indicate further that significant interactions of cultivar, drought and *V. dahliae* stresses were also obtained. One of the most common suite of traits that plants utilize to circumvent the stressful environments is the reduced stem/root ratio, thus optimizing the use of their available resources (Chapin et al., 1993; Gargallo-Garriga et al., 2014; Koevoets et al., 2016; Buhtz et al., 2017; Pandey et al., 2017). Even though, this was not shown in the double interaction, there was a significant difference in the cultivars effect (as single factor) and in the triple interaction. Our results demonstrated a significantly higher root/shoot length ratio of EMI plants compared to Skoutari plants during the stress, indicating that EMI alters its morphological traits in order to circumvent more efficiently the combination of stressors.

Stressful environments, including drought, pathogen infection and their combination, greatly influence the process of photosynthesis in most plants by altering several photosynthetic parameters such as WUE and stomatal regulation (Ashraf and Harris, 2013; Pandey et al., 2017). One of the key mechanisms of plants in order to adapt to multiple stresses is stomatal regulation by allowing plants to make the optimum CO_2_ assimilation toward evapotranspiration. In several cases of combined stresses (either abiotic-abiotic or abiotic-biotic) photosynthesis rate (*P*_N_), and leaf gas exchange parameters (g_s_, *E*, and *C*i) dramatically decreased under such conditions compared to single stress (Choi et al., 2013; Suzuki et al., 2014). In our study, the highest reduction of the photosynthetic rate and of leaf gas exchange parameters (**Figure 2**) was shown when plants were subjected either to severe drought or to combined stress, suggesting that the decline of photosynthesis rate is due to stomatal closure. Additionally, WUE and *P*_N_/g_s_ ratios were mainly dependent on water stress alone and to combined stress. Xu et al. (2008) demonstrated that when virus infection preceded water stress, plants responded better to the two stressors due to partial stomatal closure and lower transpiration rates. Our results highlighted that both varieties maintained their photosynthetic activity during the combined stress. Moreover, WUE and Pn/g were higher under combined stress than biotic stress alone (**Table 3**) The maintenance of plants photosynthetic activity is very important for their acclimation to a combination of pathogen and drought stress (Pandey et al., 2015).

During abiotic and/or biotic stress interactions, plants produce rapidly and transiently ROS, functioning as signaling molecules (i.e., induce production of ROS scavengers) and being one of their first defense responses against stresses. Likewise, fungi infection would result in oxidative stress in plants, and any concomitant stress could create inevitably additional production of ROS (Dikilitas et al., 2016). Continuous stress can cause an accumulation of ROS at the plasma membrane, producing an oxidative stress that leads to increased membrane damage and MDA content (Hasanuzzaman et al., 2013; Schieber and Chandel, 2014; Choudhury et al., 2017). Oxidative stress is often associated with excessive concentrations of H_2_O_2_ that is considered the central ROS signaling molecule (Sies, 2017). Interestingly, significant increase in H_2_O_2_ levels was monitored in all treatments compared to controls (**Tables 2** and **3**) and significant differences were observed between the two cultivars for plants under combined stress and controls (**Figures 1G,H**). The increased H_2_O_2_ levels at the end of the experiment indicated an additive effect of combined stress on H_2_O_2_ formation and a prolonged oxidative stress for the plants of Skoutari cultivar (**Figure 3**). Similarly, the triple interaction of the cultivar, *V. dahliae* and water stress had a significant combined effect on MDA one of the most well studied markers of lipid peroxidation and oxidative stress (**Figures 1I,J**). It is noteworthy that researchers have associated powerful oxidative stress with significant decrease of root growth as well (Miller et al., 2009). It should be noted that EMI cultivar exhibited better membrane homoeostasis and lower H_2_O_2_ accumulation under stress combination suggesting that may have a stronger capacity for membrane lipid replacement than Skoutari (**Figure 1**). In the review by Suzuki et al. (2014) it is clearly highlighted that the inhibition of oxidative stress can at least partially ensure plants protection against stress combination.

On the other hand, proline has been reported to be the most abundant osmolyte accumulated in response to multiple environmental stresses including water stress and pathogen infection, which acts as an ROS scavenger as well (De Ronde et al., 2000; Hanci and Cebeci, 2014; Rodziewicz et al., 2014). Recent findings have demonstrated that water stressed eggplants accumulated osmoprotectants like proline, as well as glucose and fructose and this phenomenon has been correlated with increased tolerance toward water deficit and prevention from wilting (Mibei et al., 2018). Our results showed significant difference between control plants and water stressed or *V. dahliae* infected plants in proline concentration, however, no significant difference was observed between control plants and plants under combined stress for the Skoutari cultivar. On the contrary, significant difference was observed between control plants and plants under combined stress for the EMI cultivar. This indicates that other osmoprotectants could be up-regulated during concomitant imposition of *V. dahliae* and water stress in certain eggplant cultivars.

Though the availability of genome sequences for other members of the *Solanaceae* plant family such as tomato and potato enabled gene expression and transcriptome studies for these species, no such genome sequence facilities were available for eggplant until the publication of the draft eggplant genome sequence (Hirakawa et al., 2014) and a pre-publication version from the Eggplant Genome Project^1^. Since then, various publications on comparative transcriptome analyses regarding the phylogenetic relations of eggplant and closely related wild species (Yang et al., 2014, 2015), the determination of molecular markers (Gramazio et al., 2016), characterization of genes and gene families (Barbierato et al., 2016; Li et al., 2016), gene function (Na et al., 2016), or miRNAs (Yang et al., 2013), have been made. To study the combined effect of water stress and *V. dahliae* infection at gene transcriptional level, we have selected specific marker genes, after homology search in GenBank and Sol Genomics Network and designed suitable primers for quantitative real-time polymerase chain reaction (RT-qPCR). Pathogenesis related (*PR*1 and *PR*5) and lipoxygenase (*LOX*) genes had been shown to be induced by Salicylic acid (SA) and Jasmonic acid (JA), respectively, and are commonly used as biotic stress markers in gene expression studies (Aime et al., 2008; Kissoudis et al., 2017; Mahesh et al., 2017), *AREB*, and *CBF*1 are water and cold stress related markers (Hsieh et al., 2002; Wang et al., 2016), while *NAC* genes are reported to have roles in both abiotic and biotic stresses (Tweneboah and Oh, 2017). Previous studies on the validation of eggplant reference genes for RT-qPCR assays, have indicated appropriate candidates for use in quantitative gene expression studies (Gantasala et al., 2013; Zhou et al., 2014). For our study we have selected *ACTIN* as reference gene (Zhou et al., 2014).

Our gene expression study results (**Figure 4**) showed a strong upregulation of *PR*1 and *PR*5 genes by *V. dahliae* infection as well as by water stress. Interestingly, their expression was further enhanced when combined stress was applied. These results taken together with the fact that *V. dahliae* infection preceded the water stress applied, suggest a crosstalk between biotic and abiotic stress biochemical regulatory pathways and a strong influence of water stress on biotic stress plant responses at the transcriptional level. Furthermore, the relative gene expression fold changes observed in plants under combined stress compared to plants under single stress, and particularly in the EMI cultivar, suggest that the regulation of gene expression does not necessarily fall in a mode of an additive pattern, but supports a cultivar dependent tailored made regulation. The enhanced expression of *PR*1 and *PR*5 under combined stress appears tissue dependent as well. *PR*1 is strongly induced in leaves respect to roots of both cultivars, while *PR*5 has a higher induction in roots, indicating possibly a different involvement in tissue-specific gene expression in the overall plants responses to the stresses. The low relative expression levels of *LOX* in all treatments compared to the untreated control in both cultivars, in conjunction with the strong upregulation of *PR*1 and *PR*5, indicate that the plants responses to all types of stresses applied are regulated principally *via* SA. Our results also show that the high expression of *NAC* in leaves of Skoutari water stressed plants is reduced in plants under combined stress, however, not in the EMI cultivar. This supports further a crosstalk between the biochemical pathways regulating the responses to biotic and abiotic stress and indicates a cultivar dependent mechanism that masks or suppresses the *NAC* expression. The expression patterns for *AREB* and *CBF*1 suggest that these genes are principally induced/regulated by water stress, since no differences are observed in plants under combined stress.

Our study showed that Skoutari and EMI responses to combined stress are cultivar dependent. Both cultivars were also subjected to milder drought stress (50% of FW) and we found clear difference in their responses, with EMI being the more tolerant to drought stress (data not shown). It can be speculated that the morphological adaptations, the stronger capacity for membrane lipid replacement and the higher osmoprotectant accumulation of EMI enhanced its tolerance to the combined stress. On the other hand, the increased plant growth and plant tolerance to stressful environments act in an antagonistic manner. This is the case with our cultivars as well. Skoutari cultivar exhibited the higher RGR in all conditions. However, it showed less osmoprotectant accumulation and more lipid peroxidation of membranes. Consequently, improving traits such as stability of leaf gas exchange parameters, membrane homeostasis and ROS regulation in cultivars that produce higher biomass should be further explored to gain better adaptation to higher stress environments.

It is of our interest to define further, cultivar dependent molecular responses of eggplant to either *V. dahliae*, drought stress and their possible combinations by profound transcriptomic and proteomic studies. These would allow us to dissect specific molecular pathways and define eggplant key regulatory genes for theses stresses. Recently, a characterization of an indigenous eggplant cultivars and landraces collection has been performed (Ganopoulos et al., 2015). It would be interesting to define further their responses to combined *V. dahliae* and drought stress and utilize them in future breeding projects.

## Author Contributions

ET and DK were involved in the experimental design of the work, performed experiments, analyzed, and interpreted the data and wrote the paper. EM performed and interpreted statistical analysis. IP, DT, GL, DM, and IK performed experimental work. All authors approved the final version of the manuscript to be submitted for publication and agreed to be accountable for all aspects of the work in ensuring that questions related to the accuracy and integrity of any part of the work are appropriately investigated and resolved.

## Conflict of Interest Statement

The authors declare that the research was conducted in the absence of any commercial or financial relationships that could be construed as a potential conflict of interest.

## Footnotes

1 www.eggplantgenome.org, 2017
